# Influenza A Virus Weakens the Immune Response of Mice to *Toxoplasma gondii*, Thereby Aggravating *T. gondii* Infection

**DOI:** 10.3390/vetsci10050354

**Published:** 2023-05-15

**Authors:** Junpeng Chen, Xiaoli Wang, Jinxuan Li, Lingyu Sun, Xiao Chen, Ziyu Chu, Zhenzhao Zhang, Hongxia Wu, Xiaomin Zhao, Hongmei Li, Xiao Zhang

**Affiliations:** 1 Department of Preventive Veterinary Medicine, College of Veterinary Medicine, Shandong Agricultural University, Tai’an 271002, China; chenjunpeng1223@163.com (J.C.); xmzhao66@163.com (X.Z.); 2 Shandong Provincial Key Laboratory of Animal Biotechnology and Disease Control and Prevention, Shandong Agricultural University, Tai’an 271002, China; 3 Shandong Provincial Engineering Technology Research Center of Animal Disease Control and Prevention, Shandong Agricultural University, Tai’an 271002, China

**Keywords:** influenza virus, *T. gondii*, co-infections, severe morbidity

## Abstract

**Simple Summary:**

To investigate whether the influenza virus infection affects the reproduction and transmission of *Toxoplasma gondii*, we attempted to identify the specific influencing factors. In this study, we found that decreased expression levels of IL-1β, IL-6, and IL-12 in the co-infected group were associated with the early immune responses of the host against *T. gondii* type II strain (Pru), which affected the division of *T. gondii* (Pru). Moreover, the significant decrease in the CD4^+^/CD8^+^ ratio indicated a weakened long-term immune killing ability of the host against *T. gondii* (Pru) after IAV infection. In conclusion, our study showed that a mixed infection of IAV (influenza A virus) virus and *T. gondii* (Pru) co-infection was lethal in mice and that the two pathogens may act synergistically.

**Abstract:**

This study aimed to investigate the relationship between the *T. gondii* type II strain (Pru) and respiratory viral infections, specifically focusing on the co-infection with PR8 (influenza A/Puerto Rico/8/34). In this study, we found that the number of *T. gondii* (Pru) in the lungs of co-infected mice was significantly higher and lesions were more severe than those in the group infected with *T. gondii* (Pru) alone, whereas IAV (influenza A virus) copy numbers of co-infected and PR8 alone infected groups were negligible, suggesting that infection with IAV increased the pathogenicity of *T. gondii* (Pru) in mice. The invasion and proliferation assays demonstrated no significant effect of co-infection on *T. gondii* (Pru) infection or replication in vitro. To further explore the factors causing the altered pathogenicity of *T. gondii* (Pru) caused by co-infection, we found that decreased expression levels of IL-1β, IL-6, and IL-12 in the co-infected group were associated with the early immune responses against *T. gondii* (Pru), which affected the division of *T. gondii* (Pru). Moreover, the significant decrease in the CD4^+^/CD8^+^ ratio indicated a weakened long-term immune killing ability of the host against *T. gondii* (Pru) following IAV infection. In conclusion, a *T. gondii* type II strain (Pru) could not be properly cleared by the host immune system after IAV infection, resulting in toxoplasmosis and even death in mice.

## 1. Introduction

The influenza A virus (IAV) is a common pathogen in the upper respiratory tract that affects human and animal health worldwide [1]. Co-infection with multiple microbes can cause extensive airway pathology, increasing the severity of disease and the incidence of mortality. Epidemiological studies have shown that 95% and 55% of patients in the 1918 and 2009 influenza pandemics, respectively, died from secondary infections [2,3,4,5]. Indeed, infection with IAV can make the host susceptible to a variety of pathogenic microorganisms, such as *Staphylococcus aureus*, *Streptococcus pneumoniae*, and *Mycobacterium tuberculosis*, leading to deleterious consequences due to interactions between pathogens [6,7,8]. Notably, most authors used the bacterial challenge as the major event in secondary infections caused by IAV to model their study. To our knowledge, and despite the obvious clinical importance, few studies have comprehensively investigated the mechanisms linking IAV and parasitic infections.

*Toxoplasma gondii* (*T. gondii*) is an obligate intracellular parasite of the apicomplexan family that can remarkably infect, survive, and replicate in nearly all mammalian cells. Outbreaks of toxoplasmosis secondary to other microorganisms can occasionally occur. Recent evidence indicates that approximately 13,138,600 cases of HIV-infected patients were co-infected with *T. gondii* in 312 studies documented in global papers [9]. Additionally, infection with cytomegalovirus (CMV) and *T. gondii* in the brain may lead to cognitive and behavioral disorders [10]. Latent *T. gondii* infection is common in COVID-19-infected patients of varying severity [11]. Co-infection of *T. gondii* with *Mycobacterium tuberculosis* is also very common [12]; therefore, the secondary *T. gondii* infection needs to be taken seriously and further investigated.

Here, we first employed a co-infection mouse model of the influenza virus and *T. gondii* (Pru) to investigate the differences between co-infections and single infections, implying that the synergistic effects of *T. gondii* (Pru) and IAV lead to further lung damage in mice. Specifically, we tested the immune regulatory pathways associated with the *T. gondii* (Pru) infection after IAV-induced in vivo pathology. Altogether, our study highlighted the ability of IAV to promote further *T gondii* infection by interfering with host resistance to the pathogen, thereby downregulating tissue resilience to lung inflammation.

## 2. Materials and Methods

### 2.1. Parasite Strain, Viruses, Cell Lines, and Mice

The *T. gondii* type II strain (Pru) was maintained in Vero cells (African green monkey kidney cells) at 37 °C and 5% CO_2_ using cell culture flasks (NEST Biotechnology, Wuxi, China). The influenza A/Puerto Rico/8/34 (PR8, H1N1) virus was amplified in the allantoic cavities of 10-day-old specific pathogen-free (SPF) chick embryonated eggs. Viral titers were determined by TCID_50_ (50% tissue culture infectious dose) assay using MDCK cells (canine kidney cells). The 50% tissue culture infectious dose (TCID_50_) values were calculated by the Reed–Muench method [13]. An invasion assay and a proliferation assay were performed on A549 cells (an immortalized human type II alveolar epithelial cell line) that were infected with PR8 and co-infected with Pru. All cells used in this experiment were obtained from pre-retaining in liquid nitrogen at the Laboratory of Molecular Biology of Veterinary Parasites, College of Animal Science and Technology, Shandong Agricultural University.

Six-week-old female BALB/c mice were purchased from Pengyue Experimental Animal Breeding Co. Ltd., Jinan, China and were maintained under standard conditions in accordance with the regulations specified by the Administration of Affairs Concerning Experimental Animals. The mice used in this study were approved by the Ethics Committee for Animal Experiments of the Laboratory Animal Center of Shandong Agricultural University, China.

### 2.2. Animal Experimental Procedures

To induce anesthesia, each mouse was injected with isoflurane injections; mice in Group PR8 were intranasally infected with 3 × 10^4^ PFU of influenza A/Puerto Rico/8/34 (PR8, H1N1) virus on day 1. Mice in the Pru group were injected intraperitoneally on day 2 with a dose of 10^4^ Pru. An LD50 of 3 × 10^4^ PFU of influenza A/Puerto Rico/8/34 (PR8, H1N1) virus and 10^4^ Pru were observed in the mice. Mice in the co-infected group (Pru+PR8) were inoculated intranasally with the same dose of PR8 on day 1 and then were infected intraperitoneally with the same dose of Pru on day 2. Mice in the control group received equal doses of PBS in the same manner. There were five mice in each experimental group. However, when observing pathological changes in co-infected lungs, three mice per group were used.

### 2.3. Establishment of Standard Curves of Pru and PR8

The number of *T. gondii* (Pru) was counted and serial ten-fold dilutions were made from 10^8^ to 10^2^. DNA was extracted from each sample using Genfine Biotech, Beijing, Co., Ltd. The primer for absolute quantitative real-time PCR was designed from *T. gondii* (Pru) glycerol-3-phosphate dehydrogenase (B1) gene (GenBank: MN542678) using Primer 5.0 software.

The nucleotide sequence (GenBank accession no. MH785014.1) from the GenBank database was used to design the specific primer for amplifying, purifying, and inserting the nucleocapsid protein (NP) gene of the H1N1 PR8 strain into the cloning vector-PUC19 through homologous recombination. The concentration and purity of the extracted plasmids were measured using a Smart-spec 3000 spectrophotometer (Bio-Rad, Hercules, CA, USA) and the corresponding DNA copy number was calculated.

### 2.4. Quantitative Real-Time PCR

The lung tissues from each group were cryogenically ground, RNA was extracted using the SteadyPure Universal RNA Extraction Kit (Accurate Biotechnology Co., Ltd., Changsha, China), and then cDNA was synthesized using StarScript II Reverse Transcriptase (GenStar Co., Ltd., Beijing, China). The resulting cDNA samples were then subjected to qPCR using 2×RealStar Green Fast Mixture (GenStar Co., Ltd., Beijing, China). For absolute quantitative real-time PCR, the numbers of *T. gondii* (Pru) and H1N1 influenza virus copies were calculated based on the standard curves. For relative quantitative real-time PCR, the expression levels of mouse *β-Actin* were used as endogenous controls.

### 2.5. Flow Cytometry

The adipose tissues and bronchus of each group of lungs were removed. The lungs were then cut into pieces and digested in RPMI 1640 medium (VivaCell, Shanghai, China) containing 10% FBS (Sperikon Life Science & Biotechnology Co., Ltd., Chengdu, China) and collagenase VIII (250 U/mL; Sigma Aldrich, Saint Louis, MO, USA) at 37 °C for 30 min. Cell surface staining was performed by incubating the cells with the indicated antibodies on ice for 15 min. Cells were washed and analyzed on an LSR Fortessa flow cytometer (BD Biosciences, San Diego, CA, USA) using FlowJo software (BD Biosciences, San Diego, CA, USA).

To measure the percentages of CD4^+^ and CD8^+^ cells among CD3^+^ mononuclear cells, the cells were incubated with anti-mouse CD3e-FITC (1:1200, eBioscience); anti-mouse CD4-APC (1:400, eBioscience); anti-mouse CD8a-PE (1:1600, BioLegend); anti-mouse CD45R-PerCP-Cyanine5.5 (1:800, eBioscience) for 20 min at room temperature.

To measure the percentages of macrophages and dendritic cells, cells were incubated with anti-mouse I-A/I-E-PE/Cyanine7 (1:3200, BioLegend); anti-mouse F4/80-FITC (1:100, eBioscience); anti-mouse CD11b-eFluor™ 450 (1:800, eBioscience); anti-mouse CD11c-PerCP-Cyanine5.5 (1:200, eBioscience); anti-mouse Ly-6C-APC (1:800, eBioscience) for 20 min at room temperature.

### 2.6. Histological Analysis

The lungs of the mice were collected and fixed in 4% formalin buffer (Solarbio Science & Technology Co., Ltd., Beijing, China) at 4 °C for 24 h to facilitate hematoxylin and eosin (H&E) staining. Mouse lungs were then dehydrated using an alcohol gradient and vitrified with xylene. Next, the tissues were immersed in wax for 2 h and embedded in paraffin until they solidified. Slices with a thickness of 3 μm were obtained by slicing. Afterward, the slices were dewaxed in xylene, hydrated, and stained with hematoxylin and eosin (H&E). Lastly, the slices were sealed with neutral resin; histopathological alterations were observed and recorded using a microscope.

### 2.7. Indirect Immunofluorescent Assay

A549 cells were cultured in twelve-well plates and infected with the PR8 virus at a multiplicity of infection (MOI) of 0.1 for 24 h. Following this, each well was inoculated with *T. gondii* (Pru). A total of 1 × 10^5^ freshly released tachyzoites of the Pru strain invaded the A549 monolayer for 30 min, after which the cells were washed three times with PBS. The next day, the cells were fixed with 4% PFA (Solarbio Science & Technology Co., Ltd., Beijing, China) for 50 min.

Mouse anti-influenza A nucleoprotein antibody (1:200, Biorbyt) was used as the primary antibody. Rabbit anti-TgGAP45 (1:250, lab preparation) was used as the primary antibody. Fluorescein isothiocyanate (FITC)-conjugated goat anti-mouse IgG (H + L) and Cy3-conjugated goat anti-rabbit IgG (H + L) were secondary antibodies and were incubated together. The invasion efficiency was calculated by counting the ratio of the number of infected tachyzoites/total host cells in several random fields under a fluorescence microscope [14]. The proliferation efficiency was counted for tachyzoites in 100 parasitophorous vacuoles visualized under fluorescence microscopy in several random fields of view [15]. All groups were tested 3 times independently.

### 2.8. Statistical Analysis

Data were analyzed and plotted using GraphPad Prism 8 software. The survival rate was analyzed by log-rank (Mantel–Cox) test. Other data were expressed as mean ± standard deviation and analyzed by the Mann–Whitney U test. All analyses were performed with multiple comparisons, with the exception of the parasite proliferation assay and the mice virulence assay, which were analyzed by two-way ANOVA and the Gehan–Breslow–Wilcoxon test. Significance was indicated by asterisks according to the following scale: ns, *p* > 0.05; *, *p* < 0.05; **, *p* < 0.01; ***, *p* < 0.001; ****, *p* < 0.0001.

## 3. Results

### 3.1. PR8 Virus Administration Increases the Pathogenicity of Pru in Infected Mice

**Figure 1:**
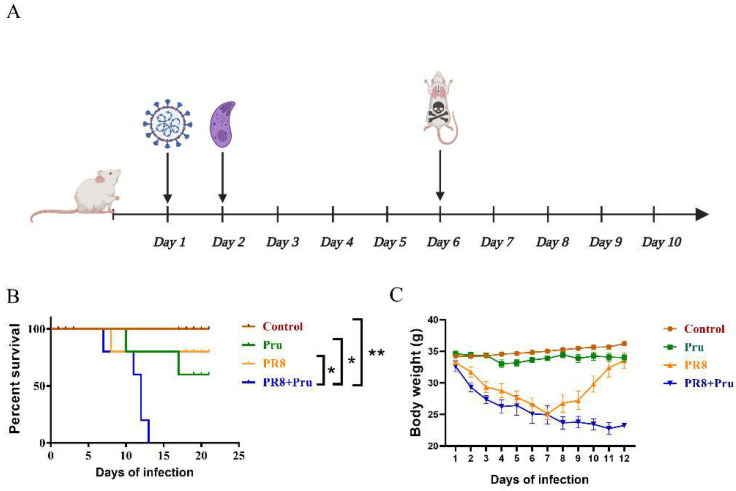
Co-infection increases mortality and reduces body weight in mice. (**A**) Pattern of mice infected with PR8 virus and T. gondii (Pru). Mice in the Pru+PR8 group were inoculated intranasally with a 3 × 104 PFU dose of PR8 on day 1, followed by infection with 10,000 doses of Pru on day 2. Mice given the same volume of PR8 on day 1 served as the PR8-infected group alone; mice given the same volume of Pru on day 2 served as the Pru-infected group alone. Non-infected mice treated with PBS in the same way served as the control group, n = 5/group. (**B**) Percent survivals of mice were monitored up to 21 days. (**C**) The body-weight curve was drawn. Mice weight changes were recorded daily. All data are representative of three independent experiments (error bars, SD). *, *p* < 0.05; **, *p* < 0.01.

Previous animal experiments have shown that individual live tachyzoites of type I strains can be lethal, while type II strains are moderately virulent and type III strains are non-virulent [16]. Therefore, we chose a moderately virulent strain of Pru type II to study the pathogenicity of co-infection in mice (Figure 1A). Mice infected with an LD_50_ dose of either the PR8 or the Pru strain alone had a low mortality rate; however, mice co-infected with PR8 and Pru had a significantly higher mortality rate, with 100% mortality, indicating a synergistic effect of the two pathogens (Figure 1B). Body weight changes in the control and the Pru-infected group fluctuated only slightly during the course of the experiment; the PR8-infected group lost weight rapidly after infection but gained weight slowly after day 8, probably due to the resistance of the body to the virus; the co-infected group lost weight consistently and showed signs of erect hair and depression, consistent with *T. gondii* (Pru) infection (Figure 1C).

### 3.2. Co-Infection Promotes T. gondii (Pru) Colonization and Aggravates Lung Pathology

**Figure 2:**
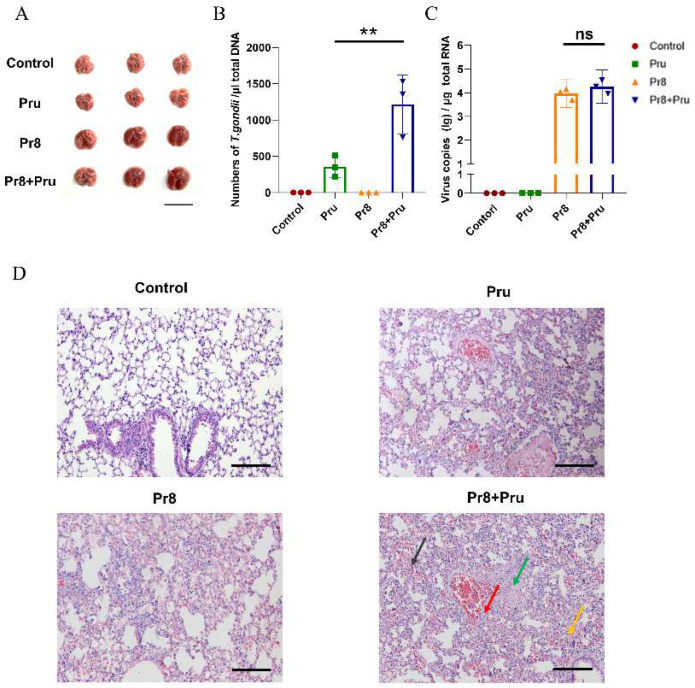
Changes in the number of *T. gondii* (Pru) and pulmonary lesions in the co-infected group. Mice were euthanized and lungs were collected on day 6 from each group; *n* = 3/group. (**A**) Representative lung images from different groups are shown, with a scale bar of 2 cm. The *T. gondii* (Pru) numbers (**B**) and viral loads (**C**) in lung tissues were determined by RT-PCR. The standard equation for Pru is y = −0.3336x + 12.043 (efficiency: 101.93%, R^2^ = 0.99311); the standard equation for PR8 is y = −0.353x + 10.747 (efficiency: 102.44%, R^2^ = 0.99428). (**D**) The histopathological examinations were performed in the lungs of infected mice on day 6 (H&E staining, 200×); scale bar, 100 μm. The orange arrow points to the area showing the loss of normal alveolar structure; the green arrow points to the area showing the disappearance of normal cellular structures; the red arrow points to the area showing the necrosis of the muscle layer; the black arrow points to the area showing alveolar hemorrhage. All unlabeled intergroup comparisons are ns. All data are representative of three independent experiments (error bars, SD). ns—not significant, **, *p* < 0.01.

The lung is an organ that is commonly susceptible to the influenza virus and *Toxoplasma.* Our results indicated that co-infected lung tissues exhibited more severe lesions in histopathological sections after 6 days of infection. (Figure 2A). To further evaluate the synergistic effects of PR8 and Pru co-infection in mice, two fluorescent quantitative PCR assays based on PR8 and Pru were established to measure viral copies and *T. gondii* (Pru) numbers in the lungs. According to standard curves, we calculated and found a significant increase in the number of *T. gondii* (Pru) in lung tissues of the co-infected group compared with PR8-infected alone (Figure 2B), while the viral loads of the co-infected group did not differ significantly from those of PR8-infected alone (Figure 2C). The histopathological examinations showed that, in the co-infected group, the lung tissues almost disintegrated, with the disappearance of normal cellular structures, muscle layer necrosis, and alveolar hemorrhage (Figure 2D). Taken together, the PR8 virus synergistically promoted lung infections and inflammatory responses caused by *T. gondii* (Pru).

### 3.3. In Vitro Invasion and Vacuoles of T. gondii (Pru) Showed No Significant Changes

**Figure 3:**
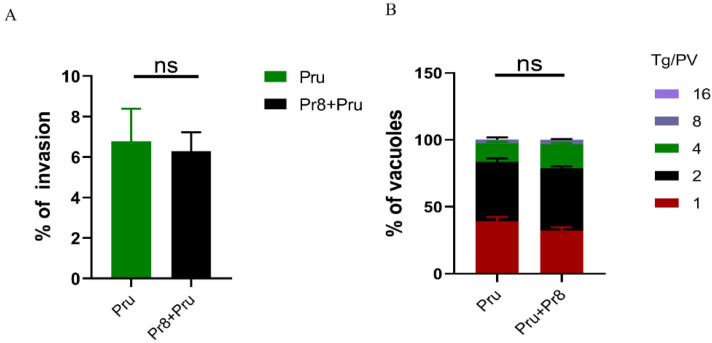
Invasion efficiency and intracellular replication assay for evaluating the proliferation of different groups in vitro. (**A**) An invasion assay and (**B**) a proliferation assay were performed on A549 cells that were infected with PR8 and co-infected with Pru. All data are representative of three independent experiments (error bars). ns—not significant.

Viability differences of *T. gondii* (Pru) were compared at the cellular level between single and co-infected groups using invasion and proliferation assays. The results showed that the cellular invasion rates of the co-infected and single-infected groups were approximately 6%, with no significant difference (Figure 3A). The statistics of *T. gondii* (Pru) division and proliferation after 24 h of growth revealed that most of the single-infected and co-infected groups were one or two divisions (Figure 3B), suggesting that co-infection had no effect on the invasion and proliferation process of *T. gondii* (Pru) in vitro.

### 3.4. Co-Infected Mice Showed Abnormal Expression of Cytokines and Abnormal Lymphocytes

**Figure 4:**
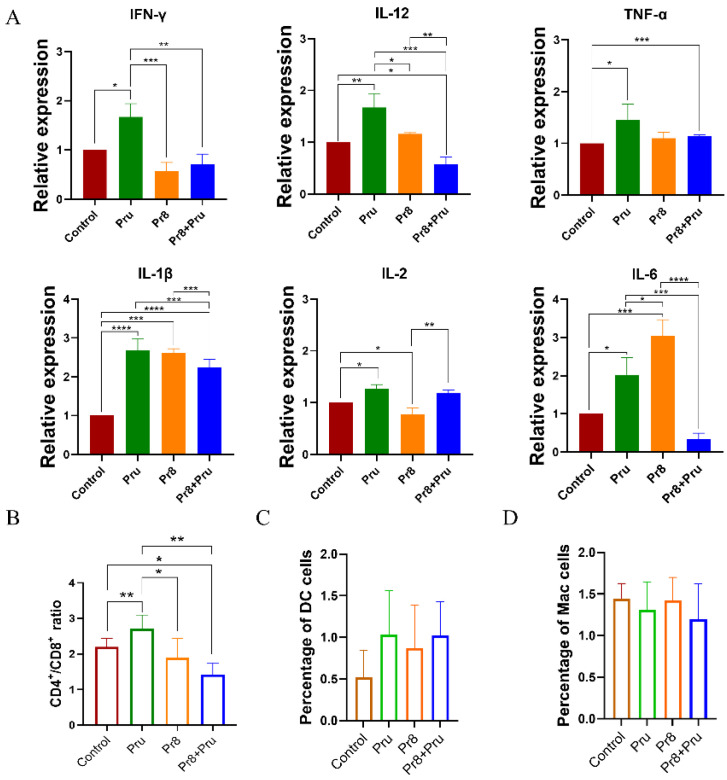
Co-infected mice exhibit differences in cytokine expression and lymphocyte proportion. Mice were euthanized and lungs were collected on day 6 from each group; *n* = 5/group. (**A**) A group of cytokines, including IFNγ, IL-12, TNFα, IL-1β, IL-2, and IL-6, were measured by qPCR on day 6. The T-lymphocytes were obtained from each group of lung tissues; the ratios of CD4^+^/CD8^+^ (**B**), DC cells (**C**), and macrophages (**D**) were measured and counted by flow cytometry. All unlabeled intergroup comparisons are ns. All data are representative of three independent experiments (error bars, SD). *, *p* < 0.05; **, *p* < 0.01; ***, *p* < 0.001; ****, *p* < 0.0001.

The invasion and proliferation of *T. gondii* (Pru) did not change significantly in vitro, but more severe pathological changes were observed in the co-infected mice. Thus, we further assessed cytokine levels to determine whether the increased severity was due to an uncontrolled inflammatory response. As shown in Figure 4A, Pru infection alone triggered significantly higher levels of IFNγ, IL-12, TNFα, IL-1β, IL-2, and IL-6 in the lungs of mice compared with the blank control. Mice infected with PR8 also exhibited higher expression levels of TNFα, IL-1β, and IL-2; this agrees with what was previously reported [17,18,19]. Nevertheless, levels of IL-12, IL-1β, and IL-6 were significantly less expressed in co-infected mice than in Pru-infected mice and were shown to be critical for the establishment and regulation of host cell-mediated immunity to the intracellular protozoan parasite *T. gondii* [20,21,22].

The co-infected group also caused changes in T-lymphocyte subsets, mainly in the form of an increased CD8^+^ T cell percentage and a decreased CD4^+^/CD8^+^ ratio compared with the control and the Pru groups, suggesting the inhibition of T cell functions (Figure 4B). These results indicated that a strong immunosuppressive response caused by IAV contributed *T. gondii* (Pru) to immune evasion.

Although the proportion of DC cells was higher in each group than in the control group, there was no significant difference nor was there a significant variability in macrophages (Figure 4C,D).

The lower expression of significantly different levels of IL-12, IL-1β, and IL-6 increased the CD8^+^ T cells and rapidly decreased the CD4^+^/CD8^+^ ratio and finally induced immune cell dysregulation, which culminated in an immunosuppressive state dominated by CD8^+^ T cells aggravating a secondary infection and subsequently facilitating the shift from a chronic to an acute infection.

## 4. Discussion

In this study, we first demonstrated that IAV infection aggravated *T. gondii* (Pru)—mediated lung damage in mice, thereby leading to higher mortality. We also verified our findings in vitro, showing that the influenza virus infection reduced the anti-parasite responses of host cells but had no effect on the viability of *T. gondii* (Pru).

Previous cases of co-infection have reported that the mortality incidence was dependent on the order in which the different pathogens were infected. Although it is clinically difficult to distinguish the order in which IAV and other pathogenic infections occur, laboratory data suggest a directional effect of co-infection when influenza viruses or other pathogens are present in different orders. Based on such reports, Kevin B. O’Brien further investigated that infecting mice with the Th1-inducing parasite *T. gondii* prior to infection with the highly pathogenic avian influenza H5N1 virus resulted in reduced lung viral titers and enhanced survival; the authors thought that live *T. gondii* may have elicited an immune response to protect mice from the influenza virus infection [23]. This study indicated that an underlying infection with the apicomplexan parasite *T. gondii* relieved the severity of the influenza virus infection in mice. The authors also found that *T. gondii* stimulated NK cells to produce IFN-γ, which regulated viral replication and thus protected the hosts from IAV infection. However, this study could not definitively rule out a role for other, as yet unidentified, cellular factors. Contrary to the above studies, our results showed that mice that were pre-infected with the H1N1 influenza virus (PR8) exhibited higher mortality and more severe lung pathology compared with mice infected with live *T. gondii* (Pru) alone. We hypothesized that this was most likely due to the different order of the two pathogens’ infections, which resulted in different immune responses being initiated by the host. Furthermore, our study found that, compared with the mice infected with IAV or *T. gondii* (Pru) alone, co-infection indeed affected cytokine changes in mice, which validated our mentioned conjecture. Similar results were found in previously reported studies. Ma et al. (2021) found that mice incubated with *Streptococcus suis (S. suis)* following IAV pre-exposure died before the virus-infected group and exhibited more severe clinical signs; conversely, when the influenza virus was administered after infection with *S. suis* infection, mortality was reduced in the infected mice compared with mice that were inoculated with the influenza virus alone [24]. Therefore, the *T. gondii* (Pru) infection can synergistically increase the pathogenicity of influenza virus in mice only if they are pre-infected with the influenza virus.

Influenza A virus (IAV) infections induce virulent pro-inflammatory immune responses characterized by ‘type I’ immunity, interferon production, and the generation of pro-inflammatory cytokines such as IL-6, TNF-α, and IL-1β. In our study, PR8 infection indeed increased the expression levels of IL-6 and IL-1β, leading to pro-inflammatory immune responses in mice. The levels of IL-12, IL-1β, and IL-6 were significantly less differentially expressed in co-infected mice than in Pru-infected mice. The *T. gondii* infection has been reported to induce an immune response in the host [25]. Not only the early induction of IL-1β was critical for establishing immunity to the *T. gondii*, but an increase or decrease in the cytokine IL-6 was also an important indicator for assessing the body’s resistance to a *T. gondii* infection. IL-6-deficient mice were associated with a failure to control parasite replication and IL-12 was required to maintain IFN-γ production in T cells [20,21,22]. These cytokine changes thus rapidly transformed a chronic *T. gondii* (Pru) infection into acute toxoplasmosis; the reduced host resistance to the *T. gondii* (Pru) infection promoted *T. gondii* (Pru) -related intracellular proliferation and facilitated parasite survival in the host. However, IAV pre-infection did not promote *T. gondii* (Pru)-mediated invasion or proliferation in in vitro studies. These results led us to suspect that the increased *T. gondii* (Pru) stored in the lung tissues of the co-infection groups in animal studies was related not only to the changes in cytokines initiated by host immune responses but also to the inhibition of T lymphocytes.

We then reported that the increase in the *T. gondii* (Pru) infection was accompanied by a decrease in the CD4^+^/CD8^+^ ratio of the lung tissues in the co-infected group compared with the Pru-infected group. A study using a toxoplasma vaccine strain in an infection model demonstrated that both CD4^+^ T cells and CD8^+^ T cells were important for controlling the *T. gondii* infection [25]. Another study indicated that the perforin-dependent cytotoxic ability of CD8^+^ T cells was involved in restricting the parasite to a state of chronic infection [26]. Although CD8^+^ T cells play an important effector role in controlling chronic infection, their maintenance is dependent on the critical help provided by CD4^+^ T cells. CD4^+^ T cells are critical for the induction of primary CD8^+^ T cell response [27]. If there are no CD4^+^ T cells, helpless CD8^+^ T cells upon re-stimulation undergo activation-induced cell death and memory response is severely impeded [28]. CD8^+^ T cell immunity generated in the absence of CD4^+^ T cells cannot be maintained and responds poorly to secondary challenges [29]. *T. gondii* induces a strong CD4^+^ T cell response, which is a major source of IFNγ during acute and chronic infections. The effector CD8^+^ T cells are one of the important sources of IFNγ, which is responsible for controlling both acute and chronic phases of infection [30]. Due to the acute infection caused by IAV in our study, we therefore speculated that the decreased CD4^+^ T cells in the co-infected group contributed to less IFNγ production, thereby leading to the diminished killing effects of CD8^+^ T cells against secondary *T. gondii* infections. Our results showed that an increase in CD8^+^ T cells and a rapid decrease in the CD4^+^/CD8^+^ ratio led to immune-cell dysregulation. This culminated in an immunosuppressive state dominated by CD8^+^ T cells, which allowed *T. gondii* to proliferate rapidly and achieve persistent infection. Taken together, our results suggested that a co-infection of *T. gondii* (Pru) and IAV promoted the proliferation of *T. gondii* (Pru) in vivo mainly due to the host immunosuppression caused by co-infection, leading to persistent infection.

We suspected that an influenza virus infection affected the innate immune response induced by *T. gondii* (Pru) in mice. With the onset of an influenza virus infection, several processes occurred that could affect the anti-bacterial innate immune response, which rendered both the upper airways and the lungs susceptible to subsequent bacterial infiltration, leading to increased bacterial load and mortality. These processes included the inhibition of type I interferons (IFNs) and the depletion of alveolar macrophages, which were associated with the host innate immunity [31]. Thus, although the inflammatory response triggered by IAV was alleviated due to the *T. gondii* (Pru) infection in mice, it was actually the IAV that reduced the host innate immune response against *T. gondii* (Pru), leading to reduced tolerance in mice.

## 5. Conclusions

In summary, a decrease in IL-6 resulted in the host being unable to control the proliferation of *T. gondii* (Pru) and a decrease in IL-12 led to a reduction in IFN-γ, the most important cytokine for killing *T. gondii* (Pru). Additionally, during co-infection with PR8 and *T. gondii* (Pru), a decrease in CD4^+^ T cells led to a reduction in CD8^+^ T cell function, which prevented the *T. gondii* (Pru) infection from becoming chronic. These results suggested that the mixed infection of IAV and *T. gondii* (Pru) was lethal in mice, with the two pathogens potentially acting synergistically to promote *T. gondii* (Pru) replication in the lungs.

## Data Availability

All data generated or analyzed during this study are included in this published article.
